# Antibiotic Alternatives: Multifunctional Ultra-Small Metal Nanoclusters for Bacterial Infectious Therapy Application

**DOI:** 10.3390/molecules29133117

**Published:** 2024-06-30

**Authors:** Yuxian Wang, Meng Gu, Jiangyang Cheng, Yusong Wan, Liying Zhu, Zhen Gao, Ling Jiang

**Affiliations:** 1 College of Food Science and Light Industry, Nanjing Tech University, Nanjing 211816, China; yxwang@njtech.edu.cn (Y.W.);; 2 College of Biotechnology and Pharmaceutical Engineering, Nanjing Tech University, Nanjing 211816, Chinagaozhen@njtech.edu.cn (Z.G.); 3 School of Chemistry and Molecular Engineering, Nanjing Tech University, Nanjing 211816, China; zlyhappy@njtech.edu.cn; 4 State Key Laboratory of Materials-Oriented Chemical Engineering, College of Food Science and Light Industry, Nanjing Tech University, Nanjing 211816, China

**Keywords:** antibiotic alternatives, nanoantibiotics, metal nanoclusters, antibacterial effect, bacterial infectious therapy

## Abstract

The prevalence of major bacterial infections has emerged as a significant menace to human health and life. Conventional treatment methods primarily rely on antibiotic therapy, but the overuse of these drugs has led to a decline in their efficacy. Moreover, bacteria have developed resistance towards antibiotics, giving rise to the emergence of superbugs. Consequently, there is an urgent need for novel antibacterial agents or alternative strategies to combat bacterial infections. Nanoantibiotics encompass a class of nano-antibacterial materials that possess inherent antimicrobial activity or can serve as carriers to enhance drug delivery efficiency and safety. In recent years, metal nanoclusters (M NCs) have gained prominence in the field of nanoantibiotics due to their ultra-small size (less than 3 nm) and distinctive electronic and optical properties, as well as their biosafety features. In this review, we discuss the recent progress of M NCs as a new generation of antibacterial agents. First, the main synthesis methods and characteristics of M NCs are presented. Then, we focus on reviewing various strategies for detecting and treating pathogenic bacterial infections using M NCs, summarizing the antibacterial effects of these nanoantibiotics on wound infections, biofilms, and oral infections. Finally, we propose a perspective on the remaining challenges and future developments of M NCs for bacterial infectious therapy.

## 1. Introduction

In recent years, there has been an incessant emergence of infectious and lethal pathogenic microorganisms, including *Escherichia coli* (*E. coli*), *Staphylococcus*, *Salmonella*, *Botox*, and other pathogenic bacteria, as well as the SARS virus, Ebola virus, and Zika virus [1]. The widespread infection and transmission of these pathogenic microorganisms have posed serious threats to human safety and normal life. The global spread of COVID-19 at the end of 2019 has brought about a profound realization of this issue [2]. In 2020, the World Health Organization (WHO) published the 2019 Global Health Estimations Report, which identified the leading causes of mortality worldwide in 2019 [3]. Among them, respiratory infections ranked fourth as a cause of death due to infectious diseases, primarily caused by pathogenic bacteria, fungi, and viruses. Although some antimicrobial drugs have provided temporary solutions for human infectious diseases, prolonged usage and misuse of these drugs have led to evolutionary adaptations and mutations in pathogenic microorganisms during treatment, resulting in resistance towards one or more drugs [4]. Consequently, microbial resistance presents a significant challenge to global public health.

The development of novel antibiotics presents a viable approach to address the issue of bacterial resistance. However, it is important to acknowledge that antibiotic development is a challenging and time-consuming process that may inadvertently contribute to the emergence of more resilient bacterial strains [5]. Thus, there is an urgent need for exploration and identification of alternative antibacterial agents as substitutes for conventional antibiotics. With the development of nanotechnology, a series of materials ranging in size from 1 to 100 nm have been sequentially synthesized, exhibiting diverse shapes, sizes, and properties [6,7]. These novel attributes bestow them with unique characteristics suitable for various biomedical applications. Nano-antibacterial materials are a class of functional materials that exhibit antibacterial activity through precise manipulation of their size, surface chemistry, and structural morphology [8]. Additionally, they can serve as carriers to enhance the efficacy and safety of antibacterial drugs [8]. Examples include non-metallic nanomaterials like graphene [9] and carbon nanotubes [10], as well as metal-based nanomaterials primarily composed of gold [11], silver [12], copper [13], etc. [14,15,16]. These nanomaterials, known as “nanoantibiotics” [17], possess antibacterial properties and can serve as alternatives to conventional antibiotics. Recently, the rapid development of nanotechnology has revolutionized various aspects of human life. Nanoantibiotics, mainly composed of nano-antibacterial materials, play a pivotal role in combating bacterial infections and offer new prospects for treating human diseases.

**Figure 1:**
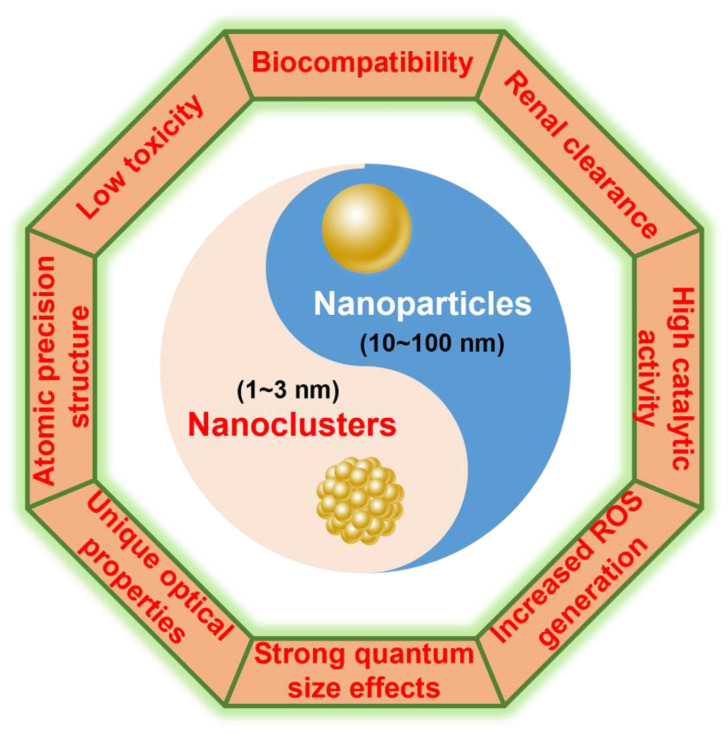
The physicochemical properties, biosafety, and bioactivity of M NCs compared to bulk M NPs.

Metal nanomaterials, including nanoparticles (NPs) and nanoclusters (NCs), find extensive applications in catalysis, sensing, and biomedical fields owing to their distinctive physical and chemical properties [18]. Metal nanoparticles (M NPs) possess unique optical, electrical, and catalytic properties, along with excellent stability within the size range of 10–100 nm [19]. Metal nanoclusters (M NCs), which consist of several or hundreds of atoms with a particle size below 3 nm, represent an intermediate structure between molecules and nanoparticles [18] (Figure 1). Compared with M NPs, M NCs exhibit unique electronic and optical properties, as well as strong quantum size effects [19,20]. It has been reported that metal nanomaterials can exert antibacterial effects on pathogenic bacteria by causing enzyme inactivation, metabolic disorders, destruction of cellular electrons, and disruption of substance transport and respiratory systems [21,22]. Due to the toxic side effects of traditional metal nanoparticles on normal human cells, their application in the biomedical field is limited. However, M NCs, owing to their ultra-small molecule-like structure, can be easily cleared by the kidneys, thereby significantly reducing the toxicity and side effects of these materials [23]. Moreover, M NCs do not exhibit drug resistance in disease treatment and demonstrate excellent biosafety [24]. Xie’s group conducted comprehensive research on the design, synthesis, and performance evaluation of M NCs with different structures [25,26,27]. These M NCs demonstrate broad-spectrum antibacterial properties against various bacterial strains, thereby expanding their potential applications in biomedical and environmental engineering. In this review, we outline the development and research progress on multifunctional M NCs for bacterial infection and emphasize the mechanisms of action associated with the designed diagnostic and therapeutic agents. Finally, the challenges and perspectives of M NCs toward bacterial infection applications are discussed.

## 2. Synthesis and Properties of M NCs

**Figure 2:**
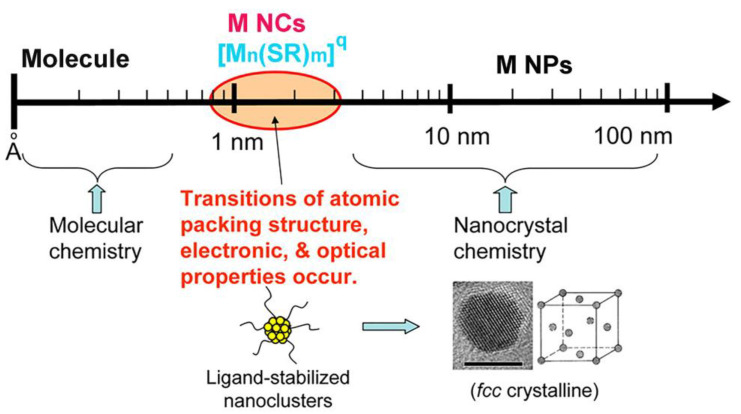
Metal nanocluster bridge between organometallic complexes and plasmonic nanoparticles. Reprinted with permission from [18]. Copyright 2024, American Chemical Society.

M NCs are nanomaterials with metal as the core and a surface modified by different organic ligands, which are transition states between atoms and nanoparticles (Figure 2) [18]. They have a precise atomic composition and structure, and ligand-protected M NCs can be represented by a “molecular formula” such as [M*_n_*(SR)*_m_*]*^q^* (where *m*, *n*, and *q* are the number of metal atoms (M), ligands (SR), and net charge in the M NC, respectively) [18]. According to different synthesis pathways, M NCs can be classified into two methods, namely “bottom-up” and “top-down” methods [28]. The former typically involves the reduction of metal ions (M^n+^) to metal atoms (M^0^) in the presence of reducing agents (such as NaBH_4_), followed by gradual nucleation and growth on the ligand surface to achieve the desired size of M NCs [29]. At present, the “bottom-up” approach is widely employed for synthesizing M NCs due to its advantages of high yield, rapid synthesis time, and straightforward reaction process. However, precise control over reduction kinetics remains crucial in achieving high-quality M NCs [30]. The “top-down” process involves the synthesis of ultra-small M NCs by utilizing ligands to etch large-sized nanoparticles, thereby necessitating a specific reaction environment to facilitate controlled etching reactions [31]. Consequently, in comparison to the “bottom-up” approach, the “top-down” method is characterized by increased time requirements and reduced yields.

**Figure 3:**
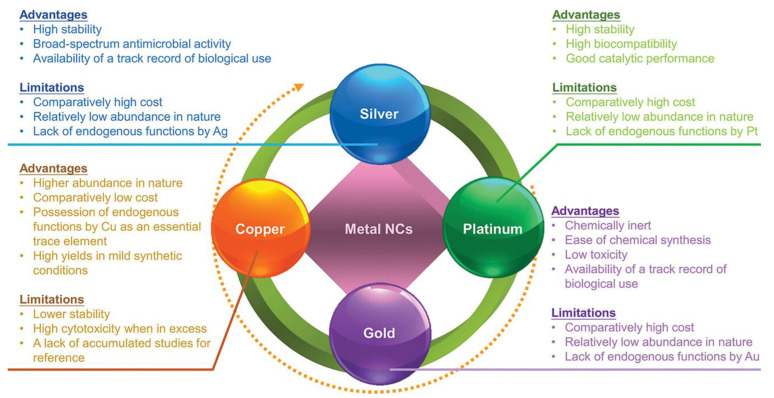
The advantages and limitations of major types of M NCs. Reprinted with permission from [38]. Copyright 2024, John Wiley and Sons.

Currently, the organic ligands commonly used for synthesizing M NCs are mainly thiol groups, because the sulfur atoms in the ligands can form strong covalent bonds with the metal atoms, which can provide good protection for M NCs [32]. Many M NCs with precise atomic numbers have been reported, such as Au NCs [33], Ag NCs [34], Cu NCs [35], and Pt NCs [36], as well as binary or multivariate nanoclusters formed by these metal complexes [37], which are widely used in various biomedical fields due to their respective advantages and disadvantages (Figure 3) [38]. For instance, Ag NCs have the advantages of high stability, broad-spectrum antimicrobial activity, and availability of a track record of biological use, but they are limited by their comparatively high cost, relatively low abundance in nature, and lack of endogenous functions of Ag. In contrast, Au NCs exhibit chemical inertness, ease of chemical synthesis, low toxicity, and availability of a track record of biological use. Nevertheless, they also face challenges such as comparatively high cost, relatively low abundance in nature, and lack of endogenous functions of Au [38].

## 3. Application of M NCs in Bacterial Infection

### 3.1. Detection of Pathogens by M NCs

**Figure 4:**
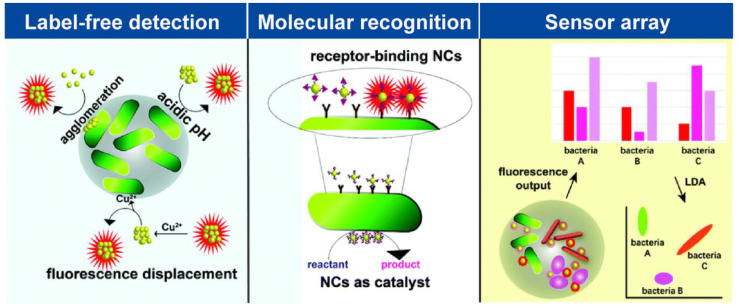
Overview of M NC-based bacterial sensing strategies. Reprinted with permission from [42]. Copyright 2024, RSC Pub.

Before taking measures for treatment after bacterial infection, accurate and rapid detection and identification of pathogenic bacteria are also particularly important. Due to the lack of timely diagnostic methods, many patients face significant challenges in effectively diagnosing drug-resistant bacteria as their condition worsens after bacterial infection. The conventional strategies for bacterial detection include plate counting, biochemical analysis, polymerase chain reaction (PCR) technology, and gene sequencing technology. However, these techniques often rely on expensive and time-consuming high-end instruments [39]. In contrast, the utilization of M NCs in biosensing has gained significant attention due to their simple synthesis process, excellent biocompatibility, strong photoluminescence properties, high photostability characteristics, and facile functionalization with other biomolecules [40]. M NCs not only enable the detection of various biomolecules, such as nucleic acids, proteins, urea glucose glutathione, etc., but also facilitate rapid and efficient identification of pathogenic bacteria through diverse strategies (Figure 4) [41,42].

#### 3.1.1. Label-Free Detection

**Figure 5:**
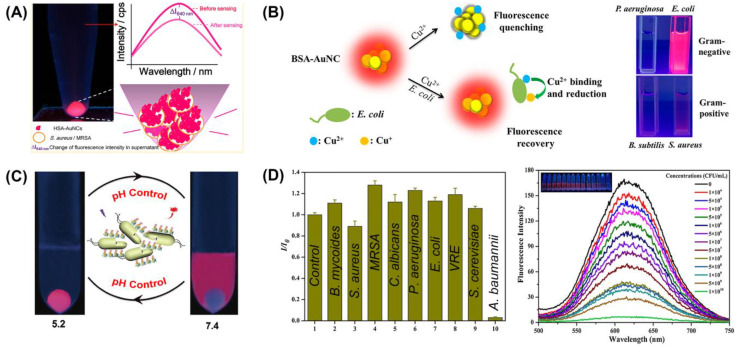
(**A**) HAS-Au NCs as a selective fluorescence probe for the detection of *S. aureus* and MRSA [45]. (**B**) Schematic illustration and images of the on–off–on Au NC-based fluorescent probe for rapid *E. coli* detection [46]. (**C**) The pH-controllable adherence of GC-Au NCs to *E. coli* cells [47]. (**D**) Highly fluorescent Au-Ag NCs recognize *A. baumannii* with high selectivity and sensitivity [48]. Reprinted with permission from [45,46,47,48]. Copyright 2024, American Chemical Society, John Wiley and Sons, and Elsevier.

Photoluminescence is one of the most important properties of M NCs, and it is also the foundation for their applications in the biomedical field. Due to the size of M NCs being comparable to the Fermi wavelength of electrons and their generation of quantum size effects, the energy levels become discontinuous and discrete, similar to those of molecules [37]. Therefore, M NCs do not exhibit the characteristic of surface plasmon resonance absorption but exhibit luminescent properties across the visible to near-infrared light spectrum. The emergence of this luminescent behavior is attributed to electronic transitions resulting from energy splitting, including both intra-band and inter-band transitions [37]. At present, an increasing number of biomolecules containing thiol groups are being employed for the synthesis of luminescent M NCs, harnessing their luminescent properties for pathogenic bacterial detection [43,44]. Chan et al. [45] employed human serum albumin (HSA)-protected gold nanoclusters (HAS-Au NCs) as fluorescent probes to detect *Staphylococcus aureus* (*S. aureus*) and methicillin-resistant *S. aureus* (MRSA). The results showed that HAS-Au NCs only exhibited a highly specific binding ability toward *S. aureus* and MRSA, leading to a substantial enhancement in fluorescence intensity (Figure 5A), which enables qualitative and quantitative analysis and detection of *S. aureus*. Yan et al. [46] developed an “on–off–on” fluorescent Au NC probe (BSA-Au NCs), which emitted a clear, red fluorescence under UV excitation (on) and quenched in the presence of Cu^2+^ (off). When the *E. coli* was present in media, the specific binding between Cu^2+^ and *E. coli* reduced the concentration of Cu^2+^ in the system, leading to the recovery of BSA-Au NC fluorescence (on) from Cu^2+^-induced quenching (Figure 5B). Applying this principle, a trace amount of *E. coli* in artificial sewage was detected within 30 min, with a lowest detectable concentration of 89 CFU/mL. In addition, Liu et al. [47] developed a rapid synthesis method of cross-linked protein-modified GC-Au NCs, which could quickly assemble into larger rod-shaped structures. Given that proteins are typical amphoteric electrolytes, the surface charge of GC-Au NCs could be regulated by environmental pH, enabling enabled enhanced adhesion between nanoclusters and *E. coli* cells (Figure 5C). Zheng et al. [48] used the coagulation effect between bacteria and M NCs to fabricate glutathione-protected Au-Ag alloy nanoclusters, wherein Ag^+^ doping significantly enhanced the photoluminescence intensity of Au NCs. The results (Figure 5D) demonstrated that the fluorescence intensity of Au-Ag NCs could be rapidly quenched by selective aggregation of nanoclusters and *Acinetobacter baumannii* (*A. baumannii*) while exhibiting no impact on the other eight pathogens, achieving unlabeled detection with an LOD of 2.3 × 10^3^ CFU/mL for *A. baumannii*. The above studies can provide valuable insights for rapid analysis and detection of different pathogenic bacteria, but the interaction mechanism between bacteria and nanoclusters still needs further research.

#### 3.1.2. Molecular Recognition

**Figure 6:**
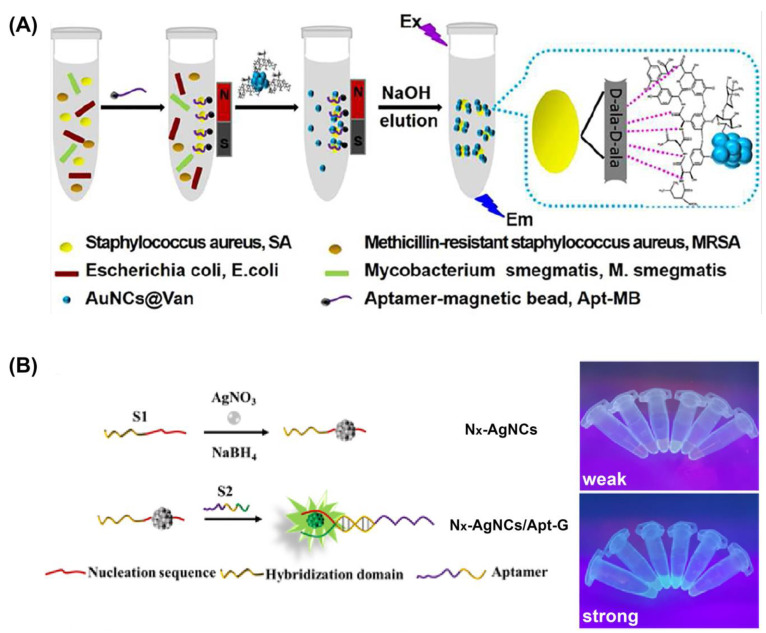
(**A**) Determination of *S. aureus* in mixtures using the aptamer–MB and AuNCs@Van dual recognition strategy [50]. (**B**) Schematic diagram and images of Apt-G enhancing the fluorescence of N*_x_*-Ag NCs [51]. Reprinted with permission from [50,51]. Copyright 2024, American Chemical Society.

The main drawback of fluorescent M NCs for bacterial detection is that their selectivity is not ideal. To overcome this challenge, a commonly employed approach involves the modification of M NCs using ligands that can recognize bacterial cell receptors [49]. Cheng et al. [50] developed a dual recognition strategy for *S. aureus* detection by designing nucleic acid aptamer-modified magnetic beads (Apt-MBs) and Au NCs protected by vancomycin (Van) (Au NCs@Van) (Figure 6A). It was demonstrated that Van could specifically identify *S. aureus* among four different pathogenic bacteria, leading to fluorescence generation upon interaction, followed by specific binding of the adapter on the magnetic bead to the bacterial membrane. Under the driving force of the magnetic field, the beads separated from other bacteria and recovered the fluorescence of the M NCs through washing with NaOH. The proposed method enabled the quantitative detection of *S. aureus* concentration in milk and human serum with high selectivity and a low LOD (70 CFU/mL). Yang et al. [51] designed fluorescent Ag NCs (N*_x_*-Ag NCs/Apt-G) with both detection and bactericidal functions, which utilized DNA as a template and were modified with specific recognition aptamers toward *S. aureus*. As shown in Figure 6B, N*_x_*-Ag NCs/Apt-G exhibited pronounced fluorescence intensity upon excitation at 365 nm, which diminished when the adapter on the nanoclusters’ surface interacted with *S. aureus* through specific binding, leading to separation of the adapter from the M NCs. Simultaneously, the M NCs exhibited significant bactericidal effects on both Gram-negative and Gram-positive bacteria, demonstrating broad-spectrum antibacterial activity. Researchers have also explored the utilization of antimicrobial peptides [52], signaling molecules [53], enzymes [54], etc., for the modification of M NCs to enable bacterial detection through recognition interactions between these biomolecules and bacteria.

#### 3.1.3. Sensor Array

**Figure 7:**
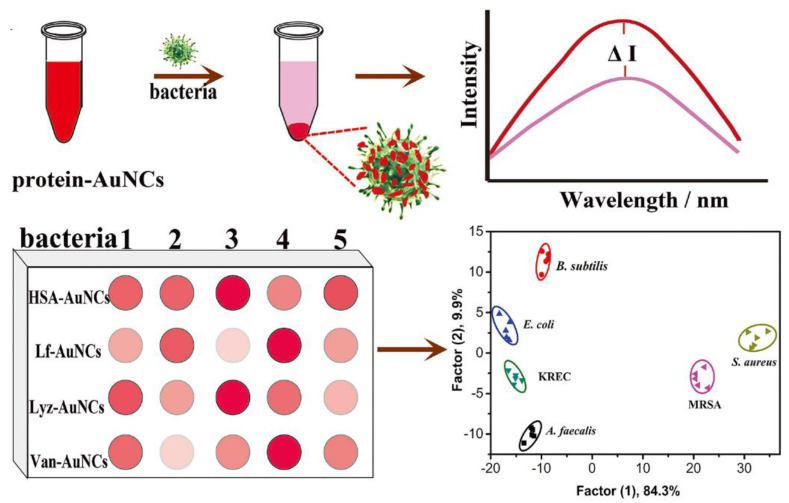
Schematic illustration of protein–Au NC-based fluorescence sensor array for discrimination of bacteria. Reprinted with permission from [55]. Copyright 2024, John Wiley and Sons.

The aforementioned methods are all designed for the detection of a single type of bacteria, whereas clinical infections often involve multiple pathogenic bacteria. Therefore, there is a need to develop sensors capable of simultaneously detecting different types of bacteria. The array-based “chemical nose/tongue” sensing method employs the collective signal characteristics generated by multiple non-selective probes to differentiate analytes, thereby enabling the detection of diverse bacterial species [55]. Ji et al. [55] synthesized four fluorescent Au NCs using human serum albumin (HSA), lysozyme (Lyz), lactoferrin (Lf), and vancomycin-modified human serum albumin (Van) as templates. These four biomolecules have a certain affinity for peptides, receptors, and polysaccharides on the surface of bacteria, which is related to the charge, hydrophobicity, and hydrophilicity of M NCs. The differences in affinity between different bacteria and various Au NCs were used to distinguish and classify multiple bacteria. As shown in Figure 7, by measuring the change in fluorescence in the supernatant after the interaction of Au NCs with bacteria and using linear discriminant analysis, the sensor array successfully identified six bacterial species (*S. aureus*, MRSA, *E. coli*, *KREC*, *B. subtilis*, and *A. faecalis*) encompassing two drug-resistant strains with an accuracy of 93.3%. This sensor array has the advantages of easy synthesis, convenient use, and high diagnostic ability, which can provide a simple, fast, and accurate diagnosis of bacterial infections in resource-limited environments. Consequently, it holds immense potential for applications in disease diagnosis and treatment, as well as food safety and environmental monitoring.

### 3.2. The Antibacterial Effect of M NCs

**Figure 8:**
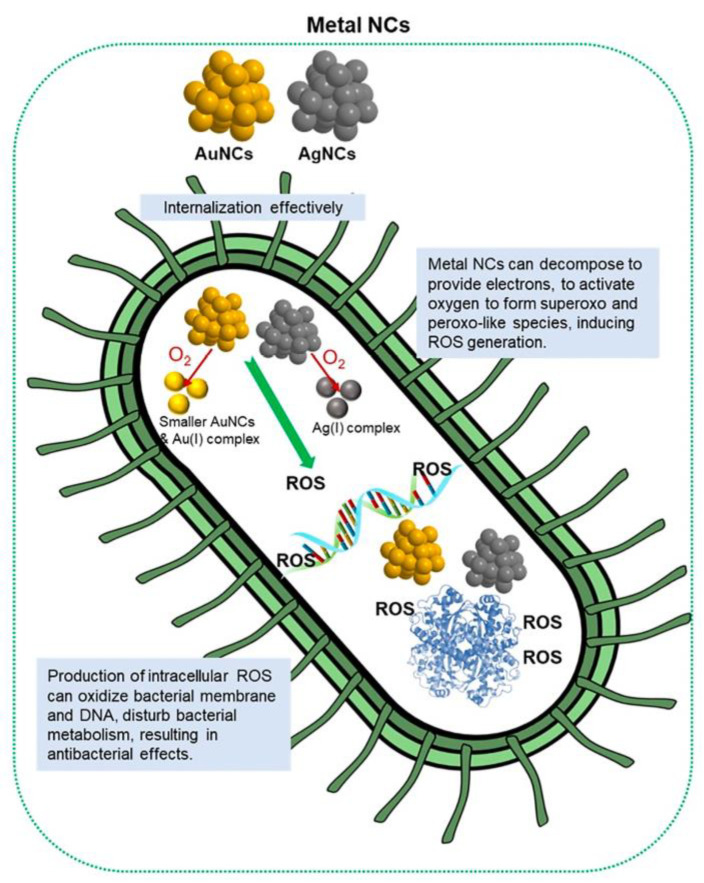
Antibacterial mechanisms of M NCs. Reprinted with permission from [58]. Copyright 2024, American Chemical Society.

Metal nanomaterials have been proven to possess significant antibacterial activity, particularly Ag nanoparticles, which primarily release metal ions to achieve bactericidal effects and do not engender bacterial drug resistance [56]. However, the particle size of these metal nanomaterials is typically large, rendering them prone to accumulation within living organisms and impeding clearance by organs such as the kidneys [57]. When metal nanomaterials are reduced to the size range of nanoclusters, not only can they be efficiently eliminated from the body, but they also exhibit enhanced antibacterial activity compared to nanoparticles [58]. This augmented antibacterial behavior is attributed to the critical internalized “size cutoff” effect (Figure 8). Ultra-small M NCs can efficiently penetrate the bacterial cell walls through their pores, facilitating internal absorption by bacteria and minimizing the interception of larger nanoparticles outside the cellular environment. On one hand, the decomposition of M NCs can generate electrons, thereby activating O_2_ and inducing the production of reactive oxygen species (ROS), which subsequently oxidize bacterial membranes. On the other hand, damaged bacterial membranes further facilitate the internalization of M NCs and their continuous accumulation, leading to interference with normal bacterial metabolism [58]. By harnessing this antibacterial mechanism, increasing research efforts have been devoted to designing multifunctional M NCs with diverse ligand modifications and the hybridization of different nanomaterials (Table 1) to address challenges associated with bacterial infections.

### 3.3. Antibacterial Infectious Therapy for M NCs

#### 3.3.1. Promoting Wound Healing and Eliminating Inflammation

**Figure 9:**
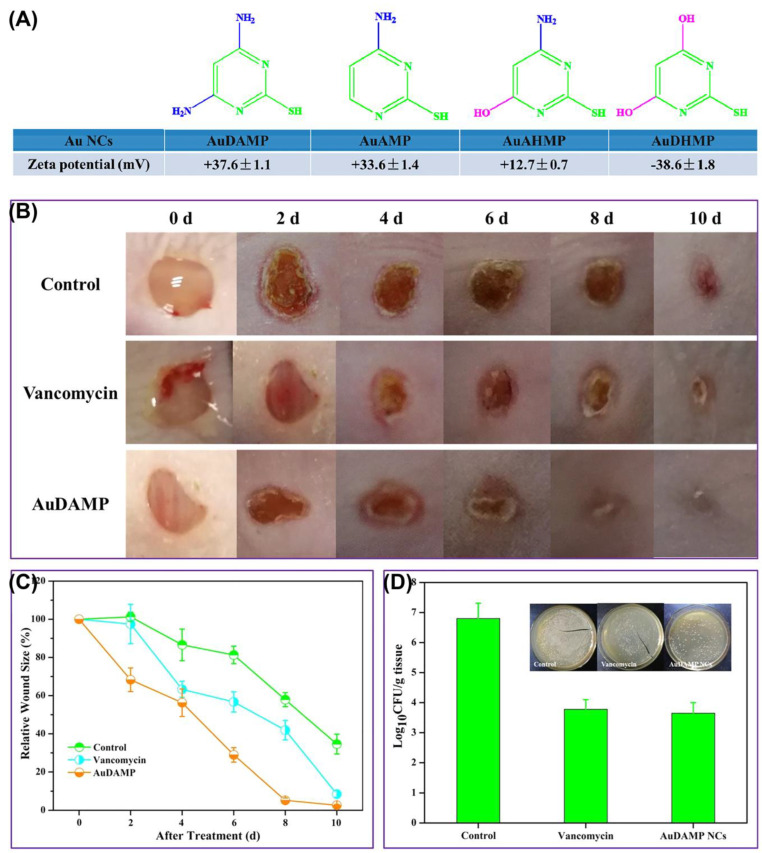
(**A**) Chemical structures of ligands and zeta potentials of Au NCs. (**B**) Representative photographs of MRSA-infected wound untreated and treated with Au NCs. (**C**) Corresponding wound sizes (relative area versus initial area). (**D**) Photographs and bacterial colonies formed on LB-agar plates. Reprinted with permission from [24]. Copyright 2024, American Chemical Society.

After skin tissue injury, the affected area is susceptible to bacterial infection. Failure to promptly address this issue can result in delayed wound healing and ulceration. M NC materials have been extensively employed to enhance the healing process of external wounds. Zheng et al. [24] developed four types of Au NCs (AuDAMP, AuAMP, AuAHMP, and AuDHMP) with thiopyrimidine as a ligand. The amino structures present in the first three ligands conferred stronger positive charges upon Au NCs and exhibited potent bactericidal effects against both Gram-negative and Gram-positive bacteria (Figure 9). In a mouse wound model infected with MRSA, the results showed that AuDAMP had a good therapeutic effect on wound healing, mainly attributed to the enzymatic activity of the Au NCs that facilitated the production of ROS. Li et al. [77] reported that M NCs could effectively alleviate the inflammatory response in wounds, thereby promoting wound healing and the proliferation and migration of related cells. In our previous work, multifunctional fluorescent Au NCs (Au_x_GSH-HHC10) with both AIE properties and antibacterial properties were prepared with glutathione (GSH) and cationic antimicrobial peptide (HHC10) [78]. They could achieve in vitro imaging of Gram-negative and Gram-positive bacteria and also exhibited broad-spectrum antibacterial effects on these two types of bacteria without developing drug resistance. The results of in vivo wound infection experiments indicated that Au_x_GSH-HHC10 could not only reduce the incidence of inflammation but also kill bacteria at the infection site and induce rapid repair of wound tissue.

#### 3.3.2. Clearing Biofilm and Preventing Its Formation

**Figure 10:**
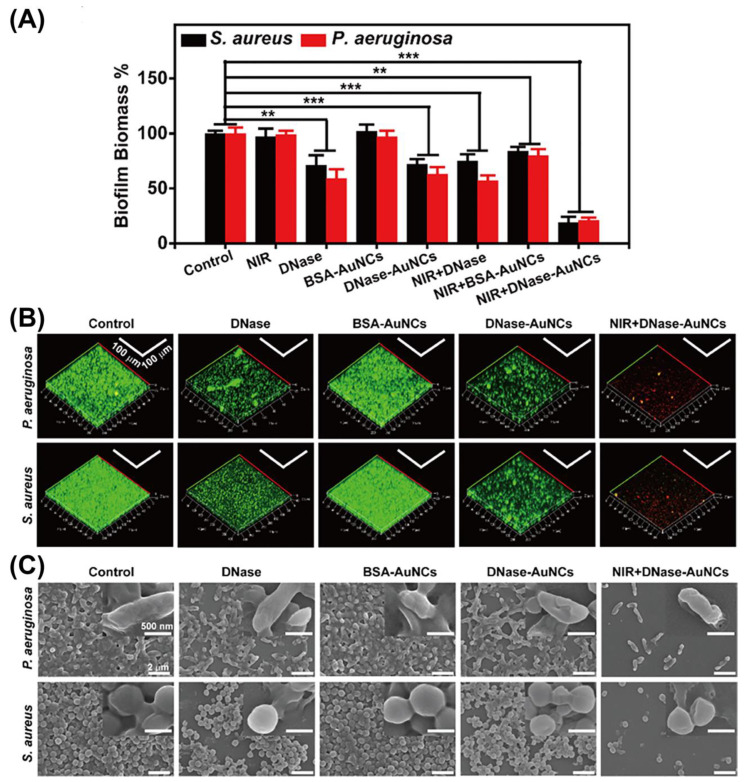
Characterization of biofilm removal effects of various experimental groups. (**A**) The reduction of biofilm (** *p* < 0.05 and *** *p* < 0.01); (**B**) fluorescence images of biofilm; (**C**) SEM images of biofilm. Reprinted with permission from [72]. Copyright 2024, American Chemical Society.

Bacteria can aggregate and secrete an extracellular matrix consisting of DNA, polysaccharides, proteins, lipids, etc., on a specific surface to form a three-dimensional polymer network structure. These extracellular matrices facilitate bacterial adhesion and subsequent formation of microcolonies that progressively grow, mature, and develop into biofilms [79]. The formation of biofilms protects bacteria from attacks by antibiotics and the host immune system, leading to bacterial resistance to antibiotics and increasing the difficulty of treating bacterial infections in clinical practice. M NCs, due to their small size, can penetrate biofilms and avoid bacterial tolerance, making them ideal materials for anti-biofilm applications [57]. Xie et al. [72] developed DNase-functionalized gold nanoclusters (DNase–Au NCs) based on extracellular DNA, which is one of the main components of biofilms. When the nanoclusters interacted with the biofilm formed by *S. aureus* and *P. aeruginosa*, DNase could decompose the extracellular DNA matrix, thus exposing defenseless bacteria. Then, under the irradiation of near-infrared light (808 nm), Au NCs exerted photodynamic and photothermal antibacterial effects. The biofilm removal capacity was up to 80%, and the bactericidal capacity was up to about 90% (Figure 10). Similarly, Okamoto et al. [73] developed a novel photosensitizer based on Au NCs to effectively inhibit the formation of bacterial biofilms associated with oral caries. The hybrid nanomaterial (Lys-AU NCs/RB) had lysozyme (Lys)-modified Au NCs as its core and was connected with light-sensitive molecule Rose red (RB). Under the irradiation of LED light, on the one hand, Au NCs transferred their own energy to RB through energy resonance transfer so that the photosensitizer could efficiently produce singlet oxygen to kill bacteria; on the other hand, Au NCs exhibited inherent antibacterial activity, and their synergistic effect effectively eliminated bacteria prior to biofilm maturation.

#### 3.3.3. Treating Oral Bacterial Infections

**Figure 11:**
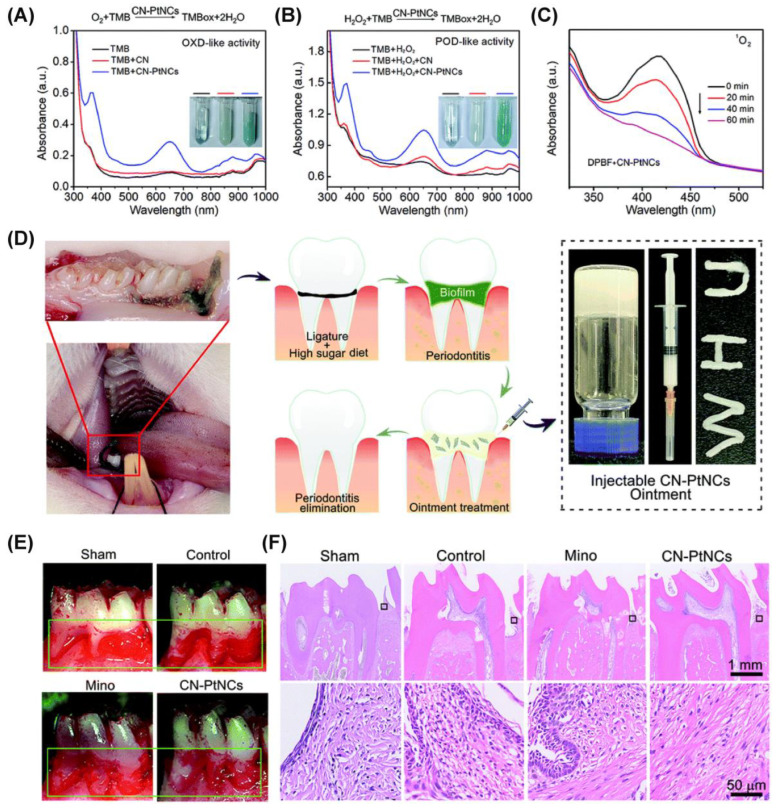
Oxidase-like and peroxidase-like properties of CN-Pt NCs and their applications in periodontitis treatment. (**A**) OXD activities of the CN and CN-Pt NCs in the presence of TMB. (**B**) POD activities of CN and CN-Pt NCs in the presence of TMB and H_2_O_2_. (**C**) The absorption intensity changes of DPBF treated by CN-Pt NCs with time. (**D**) Periodontitis model construction and use of the injectable CN-Pt NC ointment. (**E**) Fuchsin staining for biofilm (green box) recorded by stereomicroscopy. (**F**) H&E staining for the rats’ periodontal tissue. Reprinted with permission from [83]. Copyright 2024, American Chemical Society.

With the continuous popularization of oral health and hygiene, people are paying more and more attention to their oral problems. Although various diseases caused by oral bacterial infection, such as dental caries and periodontitis, are not fatal, they have high incidences and long-term characteristics [80]. Considering the remarkable contributions of nanomaterials in treating bacterial infections, researchers have initiated investigations into employing diverse nano-functional materials for the management of oral diseases [81]. However, the use of M NCs in addressing oral bacterial infections is still at an early stage of development. *Streptococcus mutans* (*S. mutans*) is a prominent pathogen responsible for dental caries. The quaternary ammonium salt-modified gold nanoclusters (QA-GNCs) developed by Xie et al. [68] not only effectively treat in vivo bacterial infections caused by MRSA but also exhibit antibacterial effects comparable to those of vancomycin at remarkably low concentrations (5 μg/mL). Coating these nanoclusters on transparent orthodontic appliances used for correcting malocclusion could efficiently prevent the formation of *S. mutans* biofilms on their surfaces [82]. In addition, the nanoclusters exhibited excellent biosafety and retained their anti-biofilm activity even after being reused more than three times within a three-month period. Wu et al. [83] modified Pt NCs with catalytic activity on g-C_3_N_4_ nanosheets (CN-Pt NCs), and the resulting nanozyme system showed dual oxidase-like and peroxidase-like activities and was used in the treatment of biofilm-induced periodontitis (Figure 11). Enzymatic property analysis revealed that CN-Pt NCs not only efficiently catalyze O_2_ to color TMB but also effectively catalyze H_2_O_2_ to color TMB, demonstrating excellent enzymatic catalytic performance. Using this enzyme property, CN-Pt NCs could effectively prevent the formation of dental plaque in the treatment of oral periodontitis and reduce inflammation and bone loss, on the other hand, showing a better therapeutic effect than clinical oral drugs (e.g., periocline).

## 4. Conclusions and Perspectives

The treatment of bacterial infections faces two major challenges, namely antibiotic resistance resulting from the overuse of antibiotics and the formation of biofilms at specific interfaces. The former creates a dilemma whereby certain superbugs cannot be treated due to a lack of effective drugs, while the latter provides a protective barrier for bacteria in the extracellular matrix, thereby reducing the efficacy of antibiotics. Consequently, it is imperative to explore novel antibacterial strategies as alternatives to traditional antibiotic therapy. In recent years, an increasing number of nanoantibiotics based on nanomaterials with antibacterial properties have been developed to address issues related to bacterial resistance and eradicate biofilms. In the present review, we provide a comprehensive overview of the synthesis methods and physicochemical properties of M NCs, with a focus on the current application status in bacterial infections, including pathogen detection and therapy. The synthesis methods of M NCs include bottom-up and top-down approaches, each with its own advantages and disadvantages. The prepared M NCs exhibit excellent optical properties, enabling them to achieve quantitative and qualitative detection and imaging of pathogens through label-free detection, molecular recognition, and the use of sensor arrays. Nanoantibiotics based on M NCs can also exert bactericidal effects on pathogens through mechanisms such as membrane damage, release of metal ions, generation of ROS, and disruption of intracellular components. In addition, M NPs can be used alone or in combination with other antibacterial agents and functional materials and are widely used to promote wound healing and eliminate inflammation, clear biofilms and prevent their formation, and treat oral bacterial infections.

Although significant progress has been made in research on M NCs as nanoantibiotics in recent years, there are still some key challenges to be addressed in the future. (1) Although numerous M NCs have been designed through atomic precision engineering, there are few types that can be used as nanoantibiotics, and their applications as traceable antimicrobial agents are still limited. (2) Although the antibacterial mechanism of M NCs has been systematically studied, the understanding of dynamic nano-biological interactions is still insufficient. (3) The effectiveness of M NCs in combating antibiotic resistance and the mechanisms to prevent resistance are issues that need to be considered in their clinical application. (4) The enzymatic-like activity of M NCs is lower compared to that of other metal materials, so constructing M NC nanozymes with high catalytic activity through atomic precision nano-chemistry will help improve the application of MNCs in disease diagnosis and therapy. (5) Although studies have reported that Au NCs can be eliminated from the body through the kidneys, residual Au NCs remain a problem in engineering designs for in vivo treatment and diagnosis, and the biological toxicity of other M NCs remains a huge challenge. We firmly believe that the collaborative efforts of scientists from diverse fields, including nanobiotechnology, material chemistry, pharmacy, and clinical medicine, hold immense potential in addressing the critical challenge posed by multidrug-resistant bacterial infections through the development of antibacterial M NCs as highly effective next-generation nanoantibiotics.

## Figures and Tables

**Table 1:** Antibacterial applications and mechanisms of different multifunctional M NCs.

System	Ligand	Formulation	Pathogens	Antibacterial Mechanism	Ref
**M NCs modified with small molecules**	Cyscein	Cys-Au NCs	*E. coli*	Intracellular ROS	[59]
		Au_25_Cys_18_	*S. aureus*	Photocatalytic generation of ROS	[60]
	p-Mercaptobenzoic acid	Au_25_ NCs; Au_102_ NCs; Au_144_ NCs	*S. aureus*	ROS; membrane damage; metabolic inactivation	[27]
	Mercaptopyrimidine	AuDAMP	*E. coli*, *MRSA*	ROS; membrane damage; DNA disruption	[24]
	Mercaptosuccinic acid	Ag NCs	*P. aeruginosa*, *A. baumannii*, *E. coli*	Enzyme-like catalysis; ROS; Ag^+^ release	[61]
	Dihydrolipoic acid	DHLA-Ag NCs	*E. coli*	Destruction of the cell membrane and fluidity; ROS; destruction the cytoplasmic membrane respiratory chain and DNA	[62]
	MUTAB	MUTAB-Au NCs	*B. Subtilis*, *S. pneumonia*, *E.coli*	Membrane damage; DNA leakage; ROS	[63]
**M NCs modified with antibacterial substances**	Daptomycin	Dap-AUDAMP NCs	*MRSA*	ROS; destruction of the cell membrane and DNA	[64]
	Bacitracin	AuNCs@Bacitracin; AgNCs@Bacitracin; CuNCs@Bacitracin	*S. aureus*	ROS; destruction of the cell membrane	[65]
	Daptomycin	Ag NCs	*S. aureus*	ROS; destruction of the cell membrane and DNA	[66]
	CCLLLLRRRRRR (Dpep)	Dpep-Ag NCs	*E. coli*, *S.aureus*	ROS; Ag^+^ release	[67]
	Quaternary ammonium (QA)	QA-Au NCs	*MRSA*	ROS; destruction of the cell membrane; membrane depolarization	[68]
	Polyethyleneimine (PEI)	PEI-Ag NCs	*E. coli*	PEI penetration and sterilization; Ag^+^ release	[69]
**M NCs modified with biomacromolecules**	DNA	DNA/Ag NC	*E. coli*	Ag^+^	[70]
	Protamine (Prot)	Prot/MTU-Au NCs	*E. coli*, *MRSA*	ROS; destruction the cell membrane	[71]
	DNase	DNase-Au NCs	*E. coli*, *S. aureus*	Photothermal and photodynamic effect	[72]
**Nanocluster hybrid system**	Lysozyme (Lys)	Lys-Au NCs/RB	*S. mutans*, *E. coli*, *A. naeslundii*, *P. gingivalis*, *P. intermedia*	ROS; destruction of the cell membrane	[73]
	Mercaptosuccinic acid (MSA)	Au NCs/CS	*E. coli*, *S. aureus*	ROS; destruction of the cell membrane	[74]
	6-Mercaptohexanoic acid, cysteamine	Au NCs/Ho-GO	*E. coli*, *S. aureus*	Piercing of bacterial membranes; ROS; metabolic inactivation	[75]
	p-Mercaptobenzoic acid	GNCs-based mixed-metal metal−organic network (MM-MON)	*E. coli*, *S. aureus*	Destruction of the cell membrane	[76]
